# Real-world effectiveness of smoking cessation aids: A population survey in England with 12-month follow-up, 2015–2020

**DOI:** 10.1016/j.addbeh.2022.107442

**Published:** 2022-12

**Authors:** Sarah E. Jackson, Loren Kock, Daniel Kotz, Jamie Brown

**Affiliations:** a Department of Behavioural Science and Health, University College London, London, UK; b SPECTRUM Consortium, UK; c Institute of General Practice, Addiction Research and Clinical Epidemiology Unit, Medical Faculty of the Heinrich-Heine-University Düsseldorf, Germany

**Keywords:** Smoking cessation, Quit attempts, Cessation aid, E-cigarettes, Varenicline, Behavioural support, Nicotine replacement therapy, Abstinence, Quit success

## Abstract

**Objective:**

To examine the real-world effectiveness of popular smoking cessation aids, adjusting for potential confounders measured up to 12 months before the quit attempt.

**Methods:**

1,045 adult (≥18y) smokers in England provided data at baseline (April 2015-November 2020) and reported a serious past-year quit attempt at 12-month follow-up. Our outcome was smoking cessation, defined as self-reported abstinence at 12 months. Independent variables were use in the most recent quit attempt of: varenicline, prescription NRT, over-the-counter NRT, e-cigarettes, and traditional behavioural support. Potential confounders were age, sex, social grade, alcohol consumption, and level of dependence (measured at baseline), variables relating to the most recent quit attempt (measured at 12-month follow-up), and survey year.

**Results:**

Participants who reported using varenicline in their most recent quit attempt had significantly higher odds of abstinence than those who did not, after adjustment for potential confounders and use of other aids (OR = 2.69, 95 %CI = 1.43–5.05). Data were inconclusive regarding whether using prescription NRT, over-the-counter NRT, e-cigarettes, or traditional behavioural support was associated with increased odds of abstinence (*p* > 0.05; Bayes factors = 0.41–1.71, expected effect size OR = 1.19), but provided moderate evidence that using e-cigarettes was more likely associated with no effect than reduced odds (Bayes factor = 0.31, expected effect size OR = 0.75).

**Conclusions:**

Use of varenicline in a quit attempt was associated with increased odds of successful smoking cessation. Data were inconclusive regarding a benefit of e-cigarettes for cessation but showed use of e-cigarettes was unlikely to be associated with reduced odds of cessation. Associations between other cessation aids and cessation were inconclusive.

## Highlight

• Use of varenicline in a quit attempt was associated with increased odds of smoking cessation.
• Data were inconclusive regarding a benefit of e-cigarettes for smoking cessation.
• Use of e-cigarettes was unlikely associated with lower odds of smoking cessation.
• Associations between other aids and cessation were inconclusive.

## 1 Introduction

Cigarettes are uniquely lethal, eventually killing up to two thirds of people who smoke them without stopping (Banks et al., 2015, Reitsma et al., 2021). People live substantially longer when they stop smoking, regardless of their age when they quit – but the sooner they quit, the more years of life expectancy they gain (Doll et al., 2004, Taylor et al., 2002 Jun 1). Maximising the success of every quit attempt is therefore critically important. A large body of evidence from randomised controlled trials suggests smokers are more likely to quit successfully if they use pharmacotherapy (e.g. nicotine replacement therapy [NRT], varenicline, cytisine, bupropion) (Cahill et al., 2013, Hartmann-Boyce et al., 2018), e-cigarettes (Hartmann-Boyce et al., 2021), or behavioural support (e.g. brief physician advice, counselling, telephone support) (Hartmann-Boyce et al., 2021). However, what is observed under trial conditions does not necessarily generalise to other populations (where people who smoke may differ from those who are selected to take part in a trial) or settings (where people may not receive the same support to use their chosen cessation aid effectively). Observational data can help us understand the effectiveness of these cessation aids in the ‘real world’ (i.e. outside an experimental setting). This is essential for informing guidance for treatment providers and enabling smokers to make informed decisions.

We previously conducted a large, observational study which examined the real-world effectiveness of the most popular quitting aids used by smokers in England (Jackson, Kotz, West, & Brown, 2019). Using data from the Smoking Toolkit Study, a large survey representative of the general population, we analysed abstinence rates among smokers who reported a recent quit attempt in relation to the type of cessation aid they used. We found use of e-cigarettes and varenicline were associated with higher abstinence rates (odds ratio [OR] 1.95 and 1.82, respectively), after adjusting for use of other aids. In addition, use of prescribed NRT was associated with higher abstinence rates, but only in older smokers (OR 1.58), and use of websites only in smokers from lower socio-economic status (OR 2.20). There was little evidence of independent benefits of using other cessation aids, such as NRT bought over-the-counter, bupropion, or behavioural support. A key limitation of this study was its cross-sectional design. In order to adjust for confounding by level of dependence (i.e., smokers who are more dependent being more likely to choose more intensive cessation aids and less likely to quit successfully), we used a validated measure (Fidler, Shahab, & West, 2011) that asked participants to rate the strength of their urges to smoke at the time of the survey. This measure served as a proxy for urges to smoke at the time of the quit attempt, which seemed to be a valid assumption (Kotz, Brown, & West, 2014) but may have been less accurate for people who were abstinent at the time of the survey than those who had relapsed.

With sufficient prospective data now available within the Smoking Toolkit Study, it is possible to apply more rigorous adjustment for potential confounding by controlling for smokers’ level of cigarette dependence prior to their quit attempt. This approach has been used previously to analyse the real-world effectiveness of prescription medication in combination with specialist behavioural support, prescription medication plus brief advice, and NRT bought over-the-counter (Kotz, Brown, & West, 2014). Participants were selected if they reported being a current smoker at baseline and having made a quit attempt between baseline and 6-month follow-up. Abstinence rates at 6-month follow-up were analysed in relation to the cessation aid they used, controlling for their level of cigarette dependence reported at baseline. Results indicated that prescription medication plus either specialist behavioural support or minimal behavioural support was associated with increased odds of abstinence (OR 2.58 and 1.55, respectively), but use of NRT bought over-the-counter was associated with decreased odds (OR 0.68). Since this study was published, the tobacco control context in England has changed, e-cigarettes have become popular, the Smoking Toolkit Study has collected data on alcohol consumption (which is associated with smoking relapse and may confound estimates of effectiveness (Kahler et al., 2010 Jul 1, Weinberger et al., 2013 May, Leeman et al., 2008 Dec)), and the follow-up period has been expanded to 12 months; offering the opportunity to update and expand this analysis of the real-world effectiveness of smoking cessation aids, with a longer-term follow-up.

This study therefore used population survey data with 12-month follow-up to examine the real-world effectiveness of popular smoking cessation aids, while adjusting for key potential confounding variables measured up to 12 months prior to the quit attempt. Specifically, we aimed to address the following research question:

• Among smokers making a quit attempt in England, is use in the quit attempt of prescription medication (NRT, varenicline, or bupropion), NRT bought over-the-counter, e-cigarettes, or traditional behavioural support (one-on-one or group counselling/support), associated with increased chances of self-reported non-smoking relative to non-use of these aids, after adjusting for potential confounding by age, sex, socioeconomic position, and level of dependence (measured at baseline; up to 12 months prior to the quit attempt), characteristics of the quit attempt (measured at follow-up), and use of the other quitting aids?

## 2 Method

### 2.1 Pre-registration

Our protocol and analysis plan were pre-registered on Open Science Framework (https://osf.io/9pzrn/). We followed this pre-registered protocol without deviation.

### 2.2 Design and study population

We used similar methodology to our previous cross-sectional and prospective studies of real-world effectiveness of smoking cessation aids (Jackson et al., 2019 Sep 1, Kotz et al., 2014). Data were drawn from the Smoking Toolkit Study; an ongoing monthly survey of representative samples of adults in England since 2006 designed to provide insights into population-wide influences on smoking and cessation by monitoring trends on a range of variables relating to smoking (Fidler et al., 2011). The study uses a form of random location sampling to select a new sample of approximately 1,700 adults aged ≥ 16 years each month, who each complete the same baseline survey. Since April 2015, smokers have been invited to participate in a follow-up survey via telephone at 12 months. The follow-up survey is intended to be representative, with efforts made to follow up all those who report smoking at baseline and consent to be recontacted, but response rates are typically low. Up to eight recontact attempts are made for each participant, at different times of day across weekdays and weekends.

For the present study, we used aggregated data from respondents to the baseline survey in the period from April 2015 to November 2020 (the most recent wave with 12-month follow-up data available at the time of analysis). We restricted our sample to respondents aged ≥ 18 years, because data have not been collected from 16 and 17-year-olds since March 2020.

Our sample comprised participants who reported:

(i) smoking cigarettes (including hand-rolled) or any other tobacco product (e.g., pipe or cigar) daily or occasionally at the time of the baseline survey (‘current smokers’);
(ii) having made at least one quit attempt between baseline and 12-month follow-up, assessed at 12-month follow-up with the question “*How many serious attempts to stop smoking have you made in the past 12 months? By serious I mean you decided that you would try to make sure you never smoked again*.”;
(iii) the success of the most recent quit attempt at 12-month follow-up (see below for definition).

### 2.3 Measures

Our outcome was self-reported continuous abstinence from the start of the most recent quit attempt reported at 12-month follow-up up to the time of the 12-month follow-up survey. Participants were asked “*How long did your most recent quit attempt last before you went back to smoking?*” Responses were coded 1 for those who responded that they were still not smoking and 0 otherwise. We note that our outcome was abstinence from smoking rather than abstinence from nicotine, so participants who reported using an e-cigarette in their quit attempt were considered abstinent if they reported that they were still not smoking tobacco at follow-up regardless of whether they were still using an e-cigarette.

Use of smoking cessation aids was assessed at 12-month follow-up with the question: *“Which, if any, of the following did you try to help you stop smoking during the most recent serious quit attempt?”* Participants were asked to indicate all that apply, and data for each of the following were coded 1 if chosen and 0 if not:

(i) prescription NRT (available in England from prescribing health professionals, including advisors at specialist stop smoking services);
(ii) NRT bought over-the-counter (NRT without a prescription);
(iii) varenicline;
(iv) bupropion;
(v) e-cigarettes;
(vi) traditional behavioural support (attended a stop smoking group or attended one or more stop smoking one-to-one counselling/advice/support session(s)). Before the Covid-19 pandemic, we would refer to this as ‘face-to-face’ behavioural support, but during the pandemic stop smoking services had to provide support virtually – usually by telephone or video calls. Thus we refer here to ‘traditional’ behavioural support to distinguish support that is traditionally delivered face-to-face from other types of behavioural support that are usually delivered remotely (e.g. telephone quitlines or websites).

For analysis, we had planned to combine variables relating to prescription medication (prescription NRT, varenicline, and bupropion) because we anticipated the numbers of participants reporting using these aids to be small (Jackson et al., 2019). We also planned to analyse data for each medication separately if samples were large enough; we were able to do this for prescription NRT and varenicline, but could not do so for bupropion due to insufficient sample size (*n* = 4).

Potential confounders included sociodemographic characteristics, alcohol consumption, and level of cigarette dependence (measured at baseline), variables relating to the most recent quit attempt (measured at 12-month follow-up), and survey year.

Sociodemographic variables were age, sex, and occupational social grade (ABC1, which includes managerial, professional and intermediate occupations, vs C2DE, which includes small employers and own‐account workers, lower supervisory and technical occupations, and semi‐routine and routine occupations, state pensioners, never worked and long‐term unemployed). This occupational measure of social grade is a valid classification that is widely used in research in UK populations (Bartley, 2016).

Alcohol consumption was assessed with Alcohol Use Disorders Identification Test (Babor, Higgins-Biddle, Saunders, Monteiro, & Organization, 2001), a 10-item screening tool developed by the WHO to assess alcohol consumption, drinking behaviours, and alcohol dependence. Scores range from 0 to 40, with higher scores indicating more problematic drinking.

Level of cigarette dependence was assessed by self-reported ratings of the strength of urges to smoke over the last 24 h (*not at all* (coded 0), *slight* (1)*, moderate* (2)*, strong* (3)*, very strong* (4)*, extremely strong* (5). This question was also coded ‘0′ for smokers who responded ‘not at all’ to the separate question “*How much of the time have you spent with the urge to smoke?*” (West, Hajek, & Belcher, 1989). This measure has been validated and performs at least as well as the Fagerström Test of Cigarette Dependence and the Heaviness of Smoking Index in predicting the outcome of cessation while not being subject to bias due to population-level changes in cigarette consumption over the time period of the study (Fidler et al., 2011).

Variables relating to the most recent quit attempt included time since the quit attempt started (less vs more than 6 months), the number of prior quit attempts in the past year (categorised as 1, 2, 3 or ≥ 4), whether the quit attempt was planned or occurred immediately when the decision to quit was made, and whether the participant cut down first or stopped abruptly.

The year of baseline survey was also included to account for changes in the availability and effectiveness of smoking cessation aids during this period (e.g. changes in e-cigarette device type).

### 2.4 Statistical analysis

Data were analysed on complete cases using SPSS v.27. We compared the baseline characteristics of eligible smokers who did vs did not respond to follow-up using *t*-tests for continuous variables and chi-square tests for categorical variables.

Bivariate associations between the use of different smoking cessation aids and potential confounders were described using *t*-tests for continuous variables and chi-square tests for categorical variables.

We then used logistic regression to analyse associations between self-reported abstinence (abstinent yes vs no) and use of different smoking cessation aids (use of a specific aid vs no use of that specific aid). Step 1 was a model including all other cessation aids (to estimate the unique association between each cessation aid and abstinence), but no potential confounders (model 1). Step 2 was a model including all potential confounders listed above, but no other cessation aids (model 2). Step 3 was a model that included all cessation aids plus all potential confounders (fully adjusted; model 3).

We calculated Bayes factors (BFs) using an online calculator (bayesfactor.info) to aid the interpretation of non-significant associations between cessation aids and abstinence in the fully adjusted model (model 3). These enabled us to examine whether the data support the alternative hypothesis (i.e. using the cessation aid is associated with increased odds of abstinence), the null hypothesis, or are insensitive. For all aids, we used a half-normal distribution, the mode at 0 (no effect), and the standard deviation equal to the expected effect size, which we set at OR = 1.19 on the basis that this is the documented difference between using no cessation support and the lowest intensity effective cessation support, according to trial evidence (Livingstone-Banks, Ordóñez-Mena, & Hartmann-Boyce, 2019). In addition, given there is debate about e-cigarettes (Wang, Bhadriraju, & Glantz, 2021) and NRT bought over-the-counter (Kotz et al., 2014) potentially suppressing quitting, we calculated a second BF for these aids with the expected effect size set at OR = 0.75 (a point in between the effect sizes observed in studies indicating lower quit rates associated with these aids among smokers motivated to quit (Kotz et al., 2014, Wang et al., 2021 Feb 1)). BFs ≥ 3 can be interpreted as evidence for the alternative hypothesis (and against the null), BFs ≤ 1/3 as evidence for the null hypothesis, and BFs between 1/3 and 3 suggest the data are insensitive to distinguish the alternative hypothesis from the null (Jeffreys, 1961, Dienes, 2014).

Following internal peer review, we added two unplanned sensitivity analyses. In the first, we coded time since the most recent quit attempt began as a 3-level (rather than binary) variable (<1 month, 1–6 months, 6–12 months) to check whether our results held when we distinguished recent quitters from those who had sustained abstinence for at least one month. In the second, we excluded participants who reported using more than one cessation aid in their most recent quit attempt from the analysis (because we did not have information about relative intensity of use of the different aids) to see if this altered the results.

Following external peer review, we added another unplanned sensitivity analysis that included additional adjustment for the timing of the Covid-19 pandemic (coded 0 for participants surveyed between April 2015 and February 2020 and 1 for those surveyed between April 2020 and November 2020 [no data were collected in March 2020]). We also added descriptive data on use of e-cigarettes and NRT at follow-up among those who used these aids to support their quit attempt.

## 3 Results

Between April 2015 and November 2020, 19,353 out of 114,078 (17.0 %) respondents to the baseline Smoking Toolkit Study survey reported being a current smoker. Follow-up data were collected 12 months later from 3,015 (15.6 %) smokers. Compared with those who did not respond, the group who completed the 12-month follow-up interview overrepresented smokers who were older, more socioeconomically advantaged, and more dependent, but there were no significant differences by sex or alcohol consumption (Supplementary Table 1).

Of the smokers who responded to follow-up, 1,104 (36.6 %) reported having made at least one quit attempt between baseline and 12-month follow-up. We excluded 59 participants with missing data on potential confounders, leaving a final sample for analysis of 1,045 participants. Characteristics of the analysed sample are summarised in Table 1. More than half of participants (57.8 %, n = 604) reported using one or more cessation aids in their most recent quit attempt. Of this group, the majority (79.3 %) reported using just one aid, 16.9 % reported using two, and 3.8 % three or more. Use of multiple cessation aids was highest among those who reported using behavioural support (81.4 %, 70/86), followed by prescription NRT (54.9 %, 56/102) and varenicline (48.1 %, 26/54). Those who reported using NRT bought over-the-counter (33.3 %, 56/168) or e-cigarettes (20.1 %, 69/344) were least likely to report using more than one aid.

**Table 1:** Characteristics of the analysed sample.

*N*				1045
**Assessed at baseline**				
	Age in years			
		18-24		12.9 (135)
		25-34		15.1 (158)
		35-44		15.7 (164)
		45-54		19.3 (202)
		55-64		18.4 (192)
		≥65		18.6 (194)
	Female			47.7 (498)
	Social grade C2DE^1^			51.0 (533)
	Alcohol consumption, mean (SD) AUDIT^2^			4.51 (5.05)
	Strength of urges to smoke, mean (SD)^3^			1.90 (1.14)
**Assessed at 12-month follow-up**				
	Currently abstinent			33.7 (352)
	Time since quit attempt began			
		<6 months		56.8 (594)
		6-12 months		43.2 (451)
	Number of quit attempts in the past year			
		1		67.4 (704)
		2		20.8 (217)
		3		7.0 (73)
		≥4		4.9 (51)
	Planned attempt			47.2 (493)
	Abrupt attempt (no cutting down first)			57.7 (603)
	Use of cessation aids^4^			
		Prescription medication^5^		14.6 (153)
			Prescription NRT	9.8 (102)
			Varenicline	5.2 (54)
			Bupropion	0.4 (4)
		NRT bought over-the-counter		16.1 (168)
		E-cigarettes		32.9 (344)
		Traditional behavioural support		8.2 (86)
		None of the above		42.2 (441)
	Number of the above cessation aids used			
		0		42.2 (441)
		1		45.8 (479)
		2		9.8 (102)
		3		1.6 (17)
		4		0.6 (6)

Note: Figures are presented as percentage (*n*), unless stated otherwise.

NRT, nicotine replacement therapy. SD, standard deviation.

^1^C2DE, more disadvantaged social grades (routine and manual occupations).

^2^AUDIT, Alcohol Use Disorders Identification Test: 0 to 40.

^3^Strength of urges to smoke: 0 (no urges) to 5 (extremely strong urges).

^4^Participants could report using more than one aid.

^5^Prescription NRT, varenicline, and bupropion combined.

Supplementary Table 2 shows associations between sample characteristics and use of the different smoking cessation aids. Use of the different aids varied by age, sex, social grade, and past quit attempts. Use of all the different cessation aids was associated with higher levels of cigarette dependence. Use of most cessation aids was associated with lower alcohol consumption (excepting varenicline and e-cigarettes), making a planned rather than unplanned quit attempt (excepting e-cigarettes), and cutting down prior to the quit date (excepting e-cigarettes and behavioural support).

Supplementary Table 3 shows associations between sample characteristics and abstinence. Compared with participants who relapsed to smoking, those who were abstinent at 12 months reported weaker urges to smoke at baseline and were less likely to have made multiple attempts to quit in the past year, planned their quit attempt, or cut down prior to the quit date.

Table 2 shows unadjusted abstinence rates and sequentially adjusted models testing associations between each cessation aid and abstinence. Overall, 33.7 % of participants (352/1,045) who reported making a quit attempt in the 12 months before the 12-month follow-up survey were abstinent at the time of the survey (median duration of abstinence: 6–12 months), ranging from 24.4 % of those who reported using NRT bought over-the-counter to support their quit attempt to 46.3 % of those who reported using varenicline.

**Table 2:** Associations between use of smoking cessation aids and abstinence.

		**Unadjusted abstinence** **% (n/N)**	**Model 1**^1^			**Model 2**^2^			**Model 3**^3^	
			**OR [95 % CI]**	***p***		**OR [95 % CI]**	***p***		**OR [95 % CI]**	***p***
No aid		35.8 (158/441)	-	-		0.85 [0.64–1.14]	0.288		-	-
Prescription medication^4^		33.3 (51/153)	0.87 [0.58–1.29]	0.487		1.42 [0.95–2.14]	0.087		1.34 [0.86–2.08]	0.196
	Prescription NRT	26.5 (27/102)	0.60 [0.37–0.98]	0.043		0.92 [0.56–1.52]	0.755		0.88 [0.52–1.51]	0.647
	Varenicline	46.3 (25/54)	1.58 [0.90–2.79]	0.113		2.82 [1.53–5.20]	0.001		2.69 [1.43–5.05]	0.002
NRT bought over the counter		24.4 (41/168)	0.57 [0.39–0.84]	0.004		0.67 [0.44–1.01]	0.058		0.71 [0.47–1.08]	0.112
E-cigarettes		32.0 (110/344)	0.86 [0.65–1.14]	0.286		1.09 [0.80–1.48]	0.580		1.12 [0.82–1.53]	0.489
Traditional behavioural support		36.0 (31/86)	1.28 [0.77–2.14]	0.337		1.27 [0.77–2.10]	0.351		1.20 [0.69–2.06]	0.519

CI, confidence interval. NRT, nicotine replacement therapy. OR, odds ratio.

Each OR and 95 % CI is for using the smoking cessation aid in question relative to not using that smoking cessation aid.

1 Model 1 = multivariable model including all smoking cessation aid variables, but no potential confounders.

2 Model 2 = multivariable model including all potential confounders (age, sex, social grade, alcohol consumption, strength of urges to smoke, time since the quit attempt started, number of prior quit attempts in the past year, whether the quit attempt was planned, whether the participant quit abruptly versus gradually, and year of the survey), but no other smoking cessation aid variables.

3 Model 3 = fully adjusted multivariable model including all potential confounders and all smoking cessation aid variables.

4 Prescription NRT, varenicline, and bupropion combined. Data are not presented separately for bupropion due to small sample size (n = 4).

Analyses that adjusted for use of other cessation aids, but no potential confounders (model 1, Table 2) indicated that participants who reported using NRT obtained on prescription or bought over-the-counter were significantly less likely to be abstinent than those who did not report using NRT. Varenicline, e-cigarettes, and traditional behavioural support were not significantly associated with abstinence after adjustment for other cessation aids.

After adjustment for sociodemographic characteristics, alcohol consumption, and level of cigarette dependence (assessed at baseline), factors relating to the quit attempt (assessed at 12 months), and survey year, but excluding other cessation aids (model 2, Table 2), the odds of abstinence were significantly higher among smokers who reported using varenicline than those who did not. There was no significant association between use of any other cessation aid and abstinence after adjusting for potential confounders. A similar pattern of results was observed when both potential confounders and use of other cessation aids were adjusted for (model 3, Table 2).

Bayes factors based on results from the fully adjusted model (model 3) indicated that the data were insensitive to detect small beneficial effects of prescription medication (combined), prescription NRT, NRT bought over-the-counter, e-cigarettes, and traditional behavioural support on abstinence (Table 3). The data were also insensitive to detect a detrimental effect of NRT bought over-the-counter, but provided moderate evidence that use of e-cigarettes was more likely associated with no effect than with reduced odds of reporting abstinence (Table 3).

**Table 3:** Bayes factors for non-significant (fully-adjusted) associations between use of smoking cessation aids and abstinence.

		**Expected effect size OR = 1.19**			**Expected effect size OR = 0.75**	
		**BF**	**Interpretation**		**BF**	**Interpretation**
Prescription medication^1^		1.71	Data are insensitive		-	-
	Prescription NRT	0.70	Data are insensitive		-	-
	Varenicline	–	–		–	–
NRT bought over the counter		0.41	Data are insensitive		2.45	Data are insensitive
E-cigarettes		1.09	Data are insensitive		0.31	Moderate evidence for H0
Traditional behavioural support		1.15	Data are insensitive		–	–

BF, Bayes factor. H0, null hypothesis (no effect).

1 Prescription NRT, varenicline, and bupropion combined.

Repeating the analyses (i) using a 3-level variable for time since the quit attempt started (<1 month, n = 113, 10.8 %; 1–6 months, n = 481, 46.0 %; 6–12 months, n = 451, 43.2 %), (ii) excluding participants who reported using more than one cessation aid (n = 125), and (iii) adjusting for the timing of the Covid-19 pandemic did not materially alter the pattern of results (Supplementary Tables 4, 5, and 6, respectively).

Of participants who reported using an e-cigarette in their quit attempt at baseline, 70.3 % (242/344) were still using an e-cigarette at 12-month follow-up (84.5 % [93/110] of those who were abstinent at follow-up and 63.7 % [149/234] of those who had relapsed to smoking; the latter indicating dual use of both tobacco and e-cigarettes). The corresponding figures for use of NRT at follow-up were 34.3 % (35/102) among those who reported using prescription NRT in their quit attempt (40.7 % [11/27] of those who were abstinent at follow-up and 32.0 % [24/75] of those who had relapsed) and 48.2 % (81/168) among those who reported using NRT bought over-the-counter (46.3 % [19/41] and 48.8 % [62/127], respectively).

## 4 Discussion

Participants who reported using varenicline in their most recent quit attempt had significantly higher odds of reporting abstinence than those who did not, after adjustment for age, sex, social grade, alcohol consumption, and level of cigarette dependence (assessed at baseline); time since the quit attempt started, number of prior quit attempts in the past year, whether the quit attempt was planned, and whether the participant quit abruptly versus gradually (assessed at 12 months); survey year; and use of other cessation aids. The data were inconclusive regarding whether use of prescription NRT, NRT bought over-the-counter, e-cigarettes, and traditional behavioural support were associated with increased odds of abstinence, but provided moderate evidence that use of e-cigarettes was unlikely to be associated with reduced odds of abstinence.

This study aimed to extend our previous cross-sectional analysis of the real-world effectiveness of popular cessation aids used by smokers in England (Jackson et al., 2019) by using a longitudinal design to better adjust for potential confounding by level of cigarette dependence prior to the quit attempt. There are similarities and differences between the two sets of results. In both studies, use of varenicline was associated with significantly increased odds of abstinence (cross-sectional OR = 1.82, longitudinal OR = 2.69) and use of behavioural support was not (OR = 1.20 and OR = 1.20, respectively), after adjustment for potential confounders and use of other cessation aids. The cross-sectional data also showed a significant association between use of e-cigarettes and increased abstinence (OR = 1.95) while the data in the present study were inconclusive (OR = 1.12, [0.82–1.53]). Similarly, there was a significant association between use of prescription NRT and increased abstinence in the cross-sectional analysis (OR = 1.34) but not in the present study (OR = 0.88).

It is not clear to what extent these possible differences are attributable to better adjustment for smokers’ pre-quit-attempt level of cigarette dependence (in which case the present estimates may be more accurate) versus the sample being less representative of smokers trying to quit (in which case the previous cross-sectional estimates may be more accurate). This study’s longitudinal design allowed us to adjust for key potential confounders such as level of cigarette dependence prior to the quit attempt. However, it relied on participants who completed the baseline survey responding to the follow-up assessment. The baseline survey recruited a large, representative sample of adults in England, and our previous cross-sectional analysis was conducted on this sample (Jackson et al., 2019). Response to follow-up was low (15.6 % of eligible participants), resulting in small samples sizes for each of the treatments evaluated. Those who responded were older and more socioeconomically advantaged, and marginally more dependent, than those who did not, limiting the generalisability of the present findings. Additionally, the quit rates among the sample followed up were higher than would be expected, which may reflect smokers who had quit being more likely to respond to the follow-up survey, some degree of forgetting failed quit attempts, or a combination of the two. If forgetting differed according to the cessation aids used in failed quit attempts, this could bias estimates of effectiveness. On the other hand, some results were inconclusive and it is possible with more data that our cross-sectional results may yet be replicated.

In the context of existing evidence, the results of the present study provide increased confidence in the real-world effectiveness of varenicline as a smoking cessation aid. A number of previous retrospective and prospective studies have shown varenicline to be effective in real-world settings (Chaiton et al., 2018, Jackson et al., 2019 Sep 1, Kaduri et al., 2015 Jun, Kotz et al., 2014 Mar, Kotz et al., 2014, Pascual et al., 2016 Jan 28); our results add to this evidence base by demonstrating a significant benefit of using varenicline even after adjusting for use of other cessation aids and relevant confounders. It is noteworthy that while varenicline was found to be associated with the highest odds of successful cessation, only 5 % of smokers who made a quit attempt reporting using it. This would appear to suggest that the benefits of varenicline should be more clearly communicated to smokers. However, in 2021 (after these data were collected), Pfizer ltd, the company that manufactures varenicline, recalled the medication as a precautionary measure due to presence of levels of *N*-nitroso-varenicline above the acceptable level of intake set by both European Medicines Agency and Medicines and Healthcare products Regulatory Agency (Medicines and Healthcare products Regulatory Agency). Although a generic version is available in some countries such as the US and Australia (Australia, 2022, Par Launches Generic of Smoking Cessation Drug Chantix [Internet]), it is not yet clear whether varenicline will return to the market in the UK; if it does not, there are alternative medications that could potentially achieve similar results. Cytisine is less well-known but has similar properties to varenicline (i.e. structurally similar to nicotine, acts as a partial agonist at nicotinic acetylcholine receptors), has been shown to be effective for smoking cessation in a number of trials (Hajek et al., 2013 Nov 1, Walker et al., 2014 Dec 18, Walker et al., 2021 Oct), and is licensed (Medicines and Healthcare products Regulatory Agency) but not yet supplied in England.

While we did not find use of e-cigarettes in a quit attempt to be significantly associated with increased odds of successful smoking cessation, the data did not show evidence of an adverse effect of e-cigarette use on smoking cessation, as has been suggested previously (Wang et al., 2021), and there remains population-level evidence that changes in the prevalence of e-cigarette use in England have been positively associated with the overall quit rates and quit success rates (Beard et al., 2016 Sep, Beard et al., 2020). We note that the majority of participants who were successful in stopping smoking with an e-cigarette were still using the e-cigarette at follow-up. The impact of ongoing e-cigarette use among ex-smokers is not yet well established: it could either reduce the risk of relapse, as it satisfies ex-smokers’ need to obtain nicotine from other sources (Notley et al., 2018 Jun 20, Notley et al., 2021), or it may increase the risk of relapse by maintaining nicotine dependence and its behavioural similarity to smoking (Everard et al., 2020 Jun 5, McMillen et al., 2019 Sep 1). Our data do not tell us anything about longer-term trajectories of e-cigarette use (i.e. beyond 12 months post-quit attempt) or what proportion of long-term users of e-cigarettes use e-cigarettes with nicotine (some may reduce the nicotine content over time). More data on the long-term effectiveness of e-cigarettes for smoking cessation are required. In addition, more evidence is needed to determine the real-world effectiveness of other cessation aids such as behavioural support and NRT obtained on prescription or bought over-the-counter.

Besides the low response rate, there were limitations associated with the available data: (i) the 12-month follow-up assessment did not capture all of the cessation aids usually assessed in the baseline Smoking Toolkit Study survey (e.g. telephone support, written self-help materials, or websites), so we were unable to examine the effectiveness of these aids; and (ii) while we controlled for a range of potential confounders, there may have been residual confounding by other variables that were not assessed, such as factors associated with self-selection of cessation aids (e.g. chronic mental or physical health conditions), the extent to which participants adhered to their chosen cessation aid, or the ways in which participants who reported using multiple cessation aids used them (e.g. concurrently or consecutively). In addition, the sample size was not large enough to explore moderation of the effectiveness of cessation aids by smokers’ characteristics. In our previous cross-sectional study, we found certain aids were more or less effective in different age groups, men vs women, more vs less advantaged social grades, and more vs less addicted smokers (Jackson et al., 2019). Finally, our study was conducted in England and results may not generalise to other countries with different tobacco control climates, availability of cessation support, and regulation of e-cigarettes.

## 5 Conclusion

In a non-representative follow-up sample of smokers in the ‘real world’, use of varenicline in a quit attempt was associated with increased odds of successful smoking cessation, after accounting for key potential confounders assessed prior to the quit attempt and use of other cessation aids. The data were inconclusive regarding a benefit of e-cigarette use for smoking cessation but showed use of e-cigarettes was not associated with reduced odds of smoking cessation. Associations between use of other cessation aids and smoking cessation were inconclusive.

## Ethics approval and consent to participate

Ethical approval for the Smoking Toolkit Study was granted originally by the UCL Ethics Committee (ID 0498/001). The data are not collected by UCL and are anonymised when received by UCL.

## Availability of data and materials

The datasets used and analysed during the current study are available from the corresponding author on request.

## Funding

Cancer Research UK funded SJ’s salary and STS data collection (C1417/A22962). The UK prevention research partnership funded LK’s salary (MR/S037519/1).

## CRediT authorship contribution statement

**Sarah E. Jackson:** Conceptualization, Methodology, Formal analysis, Investigation, Writing – original draft. **Loren Kock:** Conceptualization, Methodology, Investigation, Writing – review & editing. **Daniel Kotz:** Conceptualization, Methodology, Investigation, Writing – review & editing. **Jamie Brown:** Conceptualization, Methodology, Investigation, Resources, Writing – review & editing, Supervision, Funding acquisition.

## Declaration of Competing Interest

The authors declare the following financial interests/personal relationships which may be considered as potential competing interests: JB has received unrestricted research funding from Pfizer, who manufacture smoking cessation medications. All authors declare no financial links with tobacco companies or e-cigarette manufacturers or their representatives.
