# Supplementary material for: Real-world effectiveness of smoking cessation aids: A population survey in England with 12-month follow-up, 2015–2020
Source: Addict Behav. 2022 Dec;135:107442. doi: 10.1016/j.addbeh.2022.107442 (PMC9587352; doi:10.1016/j.addbeh.2022.107442)
Supplement: Supplementary data 1 [file mmc1.docx]

# Real-world effectiveness of smoking cessation aids: a population survey in England with 12-month follow-up, 2015-2020

Supplementary material

| **Supplementary Table 1** Baseline characteristics of smokers who did and did not respond to follow-up | | | | |
| --- | --- | --- | --- | --- |
|  | | **Responded to follow-up** | **Lost to follow-up** | ***p*** |
| *N* | | 3015 | 16338 | - |
| Age in years | |  |  |  |
|  | 18-24 | 9.0 (270) | 19.8 (3234) | <0.001 |
|  | 25-34 | 11.6 (350) | 21.8 (3557) | - |
|  | 35-44 | 13.8 (417) | 16.3 (2666) | - |
|  | 45-54 | 19.0 (573) | 16.1 (2637) | - |
|  | 55-64 | 22.1 (666) | 13.4 (2183) | - |
|  | ≥65 | 24.5 (739) | 12.6 (2059) | - |
| Female | | 46.6 (1404) | 47.2 (7709) | 0.533 |
| Social grade C2DE^1^ | | 49.6 (1492) | 59.0 (9607) | <0.001 |
| Alcohol consumption, mean (SD) AUDIT^2^ | | 4.70 (5.12) | 4.53 (5.35) | 0.093 |
| Strength of urges to smoke, mean (SD)^3^ | | 1.90 (1.14) | 1.85 (1.13) | 0.026 |
| Note: Figures are presented as percentage (*n*), unless stated otherwise.  SD, standard deviation.  ^1^C2DE, more disadvantaged social grades (routine and manual occupations).  ^2^AUDIT, Alcohol Use Disorders Identification Test: 0 to 40.  ^2^Strength of urges to smoke: 0 (no urges) to 5 (extremely strong urges). | | | | |

| **Supplementary Table 2** Associations between characteristics of the sample and use of different smoking cessation aids | | | | | | | | | | | | | |
| --- | --- | --- | --- | --- | --- | --- | --- | --- | --- | --- | --- | --- | --- |
|  | | | Prescription medication^1^ | | |  | Prescription NRT | | |  | Varenicline | | |
|  | | | Yes (*n*=153) | No (*n*=892) | *p* |  | Yes (*n*=102) | No (*n*=943) | *p* |  | Yes (*n*=54) | No (*n*=991) | *p* |
| **Characteristics assessed at baseline** | | |  |  |  |  |  |  |  |  |  |  |  |
|  | Age | |  |  |  |  |  |  |  |  |  |  |  |
|  |  | 18-24 | 5.2 (8) | 14.2 (127) | 0.001 |  | 6.9 (7) | 13.6 (128) | 0.005 |  | 3.7 (2) | 13.4 (133) | 0.190 |
|  |  | 25-34 | 11.1 (17) | 15.8 (141) | - |  | 7.8 (8) | 15.9 (150) | - |  | 14.8 (8) | 15.1 (150) | - |
|  |  | 35-44 | 13.1 (20) | 16.1 (144) | - |  | 10.8 (11) | 16.2 (153) | - |  | 14.8 (8) | 15.7 (156) | - |
|  |  | 45-54 | 23.5 (36) | 18.6 (166) | - |  | 26.5 (27) | 18.6 (175) | - |  | 24.1 (13) | 19.1 (189) | - |
|  |  | 55-64 | 18.3 (28) | 18.4 (164) | - |  | 20.6 (21) | 18.1 (171) | - |  | 14.8 (8) | 18.6 (184) | - |
|  |  | ≥65 | 28.8 (44) | 16.8 (150) | - |  | 27.5 (28) | 17.6 (166) | - |  | 27.8 (15) | 18.1 (179) | - |
|  | Female | | 50.3 (77) | 47.2 (421) | 0.474 |  | 52.9 (54) | 47.1 (444) | 0.261 |  | 46.3 (25) | 47.7 (473) | 0.837 |
|  | Social grade C2DE^1^ | | 55.6 (85) | 50.2 (448) | 0.223 |  | 60.8 (62) | 49.9 (471) | 0.038 |  | 51.9 (28) | 51.0 (505) | 0.898 |
|  | Alcohol consumption, mean (SD) AUDIT^3^ | | 3.55 (5.82) | 4.68 (4.89) | 0.010 |  | 3.61 (6.66) | 4.61 (4.84) | 0.056 |  | 4.30 (6.51) | 4.53 (4.96) | 0.745 |
|  | Strength of urges to smoke, mean (SD)^4^ | | 2.32 (1.15) | 1.83 (1.12) | <0.001 |  | 2.29 (1.17) | 1.85 (1.13) | <0.001 |  | 2.37 (1.19) | 1.87 (1.13) | 0.002 |
| **Characteristics assessed at 12-month follow-up** | | |  |  |  |  |  |  |  |  |  |  |  |
|  | Time since the quit attempt began | |  |  |  |  |  |  |  |  |  |  |  |
|  |  | <6 months | 58.2 (89) | 56.6 (505) | 0.720 |  | 54.9 (56) | 57.1 (538) | 0.677 |  | 66.7 (36) | 56.3 (558) | 0.134 |
|  |  | 6-12 months | 41.8 (64) | 43.4 (387) | - |  | 45.1 (46) | 42.9 (405) | - |  | 33.3 (18) | 43.7 (433) | - |
|  | Number of quit attempts in the past year | |  |  |  |  |  |  |  |  |  |  |  |
|  |  | 1 | 66.0 (101) | 67.6 (603) | 0.027 |  | 60.8 (62) | 68.1 (642) | 0.015 |  | 72.2 (39) | 67.1 (665) | 0.316 |
|  |  | 2 | 21.6 (33) | 20.6 (184) | - |  | 23.5 (24) | 20.5 (193) | - |  | 18.5 (10) | 20.9 (207) | - |
|  |  | 3 | 11.1 (17) | 6.3 (56) | - |  | 13.7 (14) | 6.3 (59) | - |  | 9.3 (5) | 6.9 (68) | - |
|  |  | ≥4 | 1.3 (2) | 5.5 (49) | - |  | 2.0 (2) | 5.2 (49) | - |  | 0.0 (0) | 5.1 (51) | - |
|  | Planned attempt | | 63.4 (97) | 44.4 (396) | <0.001 |  | 59.8 (61) | 45.8 (432) | 0.007 |  | 70.4 (38) | 45.9 (455) | <0.001 |
|  | Abrupt attempt (no cutting down first) | | 49.7 (76) | 59.1 (527) | 0.030 |  | 52.0 (53) | 58.3 (550) | 0.217 |  | 44.4 (24) | 58.4 (579) | 0.043 |
| Note: Figures are presented as percentage (*n*), unless stated otherwise. Data are not presented separately for bupropion due to small sample size (n=4).  NRT, nicotine replacement therapy. SD, standard deviation.  ^1^Prescription NRT, varenicline, and bupropion combined.  ^2^C2DE, more disadvantaged social grades (routine and manual occupations).  ^3^AUDIT, Alcohol Use Disorders Identification Test: 0 to 40.  ^4^Strength of urges to smoke: 0 (no urges) to 5 (extremely strong urges). | | | | | | | | | | | | | |

| **Supplementary Table 2** *continued* | | | | | | | | | | | | | |
| --- | --- | --- | --- | --- | --- | --- | --- | --- | --- | --- | --- | --- | --- |
|  | | | NRT bought over-the-counter | | |  | E-cigarettes | | |  | Traditional behavioural support | | |
|  | | | Yes (*n*=168) | No (*n*=877) | *p* |  | Yes (*n*=344) | No (*n*=701) | *p* |  | Yes (*n*=86) | No (*n*=959) | *p* |
| **Characteristics assessed at baseline** | | |  |  |  |  |  |  |  |  |  |  |  |
|  | Age | |  |  |  |  |  |  |  |  |  |  |  |
|  |  | 18-24 | 8.9 (15) | 13.7 (120) | 0.030 |  | 12.8 (44) | 13.0 (91) | <0.001 |  | 7.0 (6) | 13.5 (129) | 0.020 |
|  |  | 25-34 | 10.1 (17) | 16.1 (141) | - |  | 20.3 (70) | 12.6 (88) | - |  | 14.0 (12) | 15.2 (146) | - |
|  |  | 35-44 | 17.3 (29) | 15.4 (135) | - |  | 15.7 (54) | 15.7 (110) | - |  | 9.3 (8) | 16.3 (156) | - |
|  |  | 45-54 | 16.7 (28) | 19.8 (174) | - |  | 23.0 (79) | 17.5 (123) | - |  | 19.8 (17) | 19.3 (185) | - |
|  |  | 55-64 | 23.8 (40) | 17.3 (152) | - |  | 15.7 (54) | 19.7 (138) | - |  | 18.6 (16) | 18.4 (176) | - |
|  |  | ≥65 | 23.2 (39) | 17.7 (155) | - |  | 12.5 (43) | 21.5 (151) | - |  | 31.4 (27) | 17.4 (167) | - |
|  | Female | | 53.0 (89) | 46.6 (409) | 0.132 |  | 45.1 (155) | 48.9 (343) | 0.239 |  | 59.3 (51) | 46.6 (447) | 0.024 |
|  | Social grade C2DE^1^ | | 56.0 (94) | 50.1 (439) | 0.161 |  | 50.0 (172) | 51.5 (361) | 0.649 |  | 46.5 (40) | 51.4 (493) | 0.384 |
|  | Alcohol consumption, mean (SD) AUDIT^2^ | | 3.71 (4.15) | 4.67 (5.19) | 0.009 |  | 4.44 (5.01) | 4.55 (5.07) | 0.727 |  | 3.36 (5.60) | 4.62 (4.99) | 0.027 |
|  | Strength of urges to smoke, mean (SD)^3^ | | 2.08 (1.04) | 1.86 (1.15) | 0.017 |  | 2.10 (1.17) | 1.80 (1.11) | <0.001 |  | 2.17 (1.05) | 1.87 (1.14) | 0.018 |
| **Characteristics assessed at 12-month follow-up** | | |  |  |  |  |  |  |  |  |  |  |  |
|  | Time since the quit attempt began | |  |  |  |  |  |  |  |  |  |  |  |
|  |  | <6 months | 56.5 (95) | 56.9 (499) | 0.933 |  | 55.8 (192) | 57.3 (402) | 0.638 |  | 52.3 (45) | 57.2 (549) | 0.377 |
|  |  | 6-12 months | 43.5 (73) | 43.1 (378) | - |  | 44.2 (152) | 42.7 (299) | - |  | 47.7 (41) | 42.8 (410) | - |
|  | Number of quit attempts in the past year | |  |  |  |  |  |  |  |  |  |  |  |
|  |  | 1 | 64.3 (108) | 68.0 (596) | 0.459 |  | 66.9 (230) | 67.6 (474) | 0.598 |  | 69.8 (60) | 67.2 (644) | 0.178 |
|  |  | 2 | 22.0 (37) | 20.5 (180) | - |  | 20.3 (70) | 21.0 (147) | - |  | 22.1 (19) | 20.6 (198) | - |
|  |  | 3 | 6.5 (11) | 7.1 (62) | - |  | 8.4 (29) | 6.3 (44) | - |  | 8.1 (7) | 6.9 (66) | - |
|  |  | ≥4 | 7.1 (12) | 4.4 (39) | - |  | 4.4 (15) | 5.1 (36) | - |  | 0.0 (0) | 5.3 (51) | - |
|  | Planned attempt | | 64.3 (108) | 43.9 (385) | <0.001 |  | 46.2 (159) | 47.6 (334) | 0.664 |  | 57.0 (49) | 46.3 (444) | 0.057 |
|  | Abrupt attempt (no cutting down first) | | 49.4 (83) | 59.3 (520) | 0.017 |  | 54.4 (187) | 59.3 (416) | 0.125 |  | 55.8 (48) | 57.9 (555) | 0.711 |
| Note: Figures are presented as percentage (*n*), unless stated otherwise. Data are not presented separately for bupropion due to small sample size (n=4).  NRT, nicotine replacement therapy. SD, standard deviation.  ^1^Prescription NRT, varenicline, and bupropion combined.  ^2^C2DE, more disadvantaged social grades (routine and manual occupations).  ^3^AUDIT, Alcohol Use Disorders Identification Test: 0 to 40.  ^4^Strength of urges to smoke: 0 (no urges) to 5 (extremely strong urges). | | | | | | | | | | | | | |

| **Supplementary Table 3** Associations between characteristics of the sample and abstinence | | | | | |
| --- | --- | --- | --- | --- | --- |
|  | | | Not abstinent (*n*=693) | Abstinent (*n*=352) | *p* |
| **Characteristics assessed at baseline** | | |  |  |  |
|  | Age | |  |  |  |
|  |  | 18-24 | 13.6 (94) | 11.6 (41) | 0.256 |
|  |  | 25-34 | 14.6 (101) | 16.2 (57) | - |
|  |  | 35-44 | 15.9 (110) | 15.3 (54) | - |
|  |  | 45-54 | 20.9 (145) | 16.2 (57) | - |
|  |  | 55-64 | 18.0 (125) | 19.0 (67) | - |
|  |  | ≥65 | 17.0 (118) | 21.6 (76) | - |
|  | Female | | 48.6 (337) | 45.7 (161) | 0.377 |
|  | Social grade C2DE^1^ | | 52.4 (363) | 48.3 (170) | 0.212 |
|  | Alcohol consumption, mean (SD) AUDIT^3^ | | 4.50 (5.01) | 4.55 (5.13) | 0.875 |
|  | Strength of urges to smoke, mean (SD)^4^ | | 2.05 (1.16) | 1.60 (1.03) | <0.001 |
| **Characteristics assessed at 12-month follow-up** | | |  |  |  |
|  | Time since the quit attempt began | |  |  |  |
|  |  | <6 months | 61.2 (424) | 48.3 (170) | <0.001 |
|  |  | 6-12 months | 38.8 (269) | 51.7 (182) | - |
|  | Number of quit attempts in the past year | |  |  |  |
|  |  | 1 | 59.7 (414) | 82.4 (290) | <0.001 |
|  |  | 2 | 26.1 (181) | 10.2 (36) | - |
|  |  | 3 | 8.5 (59) | 4.0 (14) | - |
|  |  | ≥4 | 5.6 (39) | 3.4 (12) | - |
|  | Planned attempt | | 53.1 (368) | 35.5 (125) | <0.001 |
|  | Abrupt attempt (no cutting down first) | | 50.2 (348) | 72.4 (255) | <0.001 |
| Note: Figures are presented as percentage (*n*), unless stated otherwise. Data are not presented separately for bupropion due to small sample size (n=4).  NRT, nicotine replacement therapy. SD, standard deviation.  ^1^Prescription NRT, varenicline, and bupropion combined.  ^2^C2DE, more disadvantaged social grades (routine and manual occupations).  ^3^AUDIT, Alcohol Use Disorders Identification Test: 0 to 40.  ^4^Strength of urges to smoke: 0 (no urges) to 5 (extremely strong urges). | | | | | |

| **Supplementary Table 4** Associations between use of smoking cessation aids and abstinence: sensitivity analysis using 3-level variable for time since the quit attempt started (<1m, 1-6m, 6-12m) | | | | | | | | | | |
| --- | --- | --- | --- | --- | --- | --- | --- | --- | --- | --- |
|  | | **Unadjusted abstinence**  **% (n/N)** | **Model 1^1^** | |  | **Model 2^2^** | |  | **Model 3^3^** | |
|  |  |  | **OR [95% CI]** | ***p*** |  | **OR [95% CI]** | ***p*** |  | **OR [95% CI]** | ***p*** |
| No aid | | 35.8 (158/441) | - | - |  | 0.85 [0.64-1.15] | 0.290 |  | - | - |
| Prescription medication^4^ | | 33.3 (51/153) | 0.87 [0.58-1.29] | 0.487 |  | 1.43 [0.95-2.14] | 0.087 |  | 1.34 [0.86-2.08] | 0.197 |
|  | Prescription NRT | 26.5 (27/102) | 0.60 [0.37-0.98] | 0.043 |  | 0.92 [0.56-1.52] | 0.750 |  | 0.88 [0.52-1.50] | 0.639 |
|  | Varenicline | 46.3 (25/54) | 1.58 [0.90-2.79] | 0.113 |  | 2.82 [1.53-5.21] | 0.001 |  | 2.69 [1.43-5.06] | 0.002 |
| NRT bought over the counter | | 24.4 (41/168) | 0.57 [0.39-0.84] | 0.004 |  | 0.67 [0.44-1.01] | 0.057 |  | 0.71 [0.47-1.08] | 0.112 |
| E-cigarettes | | 32.0 (110/344) | 0.86 [0.65-1.14] | 0.286 |  | 1.09 [0.80-1.48] | 0.579 |  | 1.12 [0.82-1.53] | 0.488 |
| Traditional behavioural support | | 36.0 (31/86) | 1.28 [0.77-2.14] | 0.337 |  | 1.27 [0.77-2.10] | 0.351 |  | 1.20 [0.69-2.07] | 0.517 |
| CI, confidence interval. NRT, nicotine replacement therapy. OR, odds ratio.  ^1^Model 1 = multivariable model including all smoking cessation aid variables, but no potential confounders.  ^2^Model 2 = multivariable model including all potential confounders (age, sex, social grade, alcohol consumption, strength of urges to smoke, time since the quit attempt started, number of prior quit attempts in the past year, whether the quit attempt was planned, whether the participant quit abruptly versus gradually, and year of the survey), but no other smoking cessation aid variables.  ^3^Model 3 = fully adjusted multivariable model including all potential confounders and all smoking cessation aid variables.  Each OR and 95% CI is for using the smoking cessation aid in question relative to not using that smoking cessation aid.  ^4^Prescription NRT, varenicline, and bupropion combined. Data are not presented separately for bupropion due to small sample size (n=4). | | | | | | | | | | |

| **Supplementary Table 5** Associations between use of smoking cessation aids and abstinence: sensitivity analysis excluding participants who reported using more than one cessation aid in their most recent quit attempt | | | | | | | | | | |
| --- | --- | --- | --- | --- | --- | --- | --- | --- | --- | --- |
|  | | **Unadjusted abstinence**  **% (n/N)** | **Model 1^1^** | |  | **Model 2^2^** | |  | **Model 3^3^** | |
|  |  |  | **OR [95% CI]** | ***p*** |  | **OR [95% CI]** | ***p*** |  | **OR [95% CI]** | ***p*** |
| No aid | | 35.8 (158/441) | - | - |  | 0.85 [0.63-1.15] | 0.299 |  | - | - |
| Prescription medication^4^ | | 36.8 (28/76) | 1.05 [0.63-1.73] | 0.865 |  | 1.52 [0.89-2.60] | 0.125 |  | 1.61 [0.92-2.82] | 0.099 |
|  | Prescription NRT | 28.3 (13/46) | 0.71 [0.36-1.38] | 0.308 |  | 0.95 [0.47-1.93] | 0.889 |  | 1.06 [0.51-2.20] | 0.881 |
|  | Varenicline | 50.0 (14/28) | 1.79 [0.83-3.85] | 0.136 |  | 2.65 [1.16-6.07] | 0.021 |  | 2.82 [1.20-6.58] | 0.017 |
| NRT bought over the counter | | 28.6 (32/112) | 0.72 [0.46-1.13] | 0.150 |  | 0.85 [0.53-1.37] | 0.507 |  | 0.95 [0.58-1.57] | 0.846 |
| E-cigarettes | | 34.2 (94/275) | 0.93 [0.68-1.28] | 0.654 |  | 1.14 [0.82-1.59] | 0.441 |  | 1.20 [0.84-1.71] | 0.323 |
| Traditional behavioural support | | 31.3 (5/16) | 0.81 [0.28-2.39] | 0.708 |  | 0.84 [0.27-2.60] | 0.765 |  | 0.92 [0.29-2.87] | 0.880 |
| CI, confidence interval. NRT, nicotine replacement therapy. OR, odds ratio.  ^1^Model 1 = multivariable model including all smoking cessation aid variables, but no potential confounders.  ^2^Model 2 = multivariable model including all potential confounders (age, sex, social grade, alcohol consumption, strength of urges to smoke, time since the quit attempt started, number of prior quit attempts in the past year, whether the quit attempt was planned, whether the participant quit abruptly versus gradually, and year of the survey), but no other smoking cessation aid variables.  ^3^Model 3 = fully adjusted multivariable model including all potential confounders and all smoking cessation aid variables.  Each OR and 95% CI is for using the smoking cessation aid in question relative to not using that smoking cessation aid.  ^4^Prescription NRT, varenicline, and bupropion combined. Data are not presented separately for bupropion due to small sample size (n=4). | | | | | | | | | | |

| **Supplementary Table 6** Associations between use of smoking cessation aids and abstinence: sensitivity analysis adjusting for the timing of the Covid-19 pandemic | | | | | | | | | | |
| --- | --- | --- | --- | --- | --- | --- | --- | --- | --- | --- |
|  | | **Unadjusted abstinence**  **% (n/N)** | **Model 1^1^** | |  | **Model 2^2^** | |  | **Model 3^3^** | |
|  |  |  | **OR [95% CI]** | ***p*** |  | **OR [95% CI]** | ***p*** |  | **OR [95% CI]** | ***p*** |
| No aid | | 35.8 (158/441) | - | - |  | 0.87 [0.65-1.17] | 0.348 |  | - | - |
| Prescription medication^4^ | | 33.3 (51/153) | 0.87 [0.58-1.29] | 0.487 |  | 1.44 [0.96-2.17] | 0.077 |  | 1.35 [0.87-2.09] | 0.186 |
|  | Prescription NRT | 26.5 (27/102) | 0.60 [0.37-0.98] | 0.043 |  | 0.95 [0.57-1.56] | 0.826 |  | 0.90 [0.53-1.54] | 0.704 |
|  | Varenicline | 46.3 (25/54) | 1.58 [0.90-2.79] | 0.113 |  | 2.82 [1.52-5.21] | 0.001 |  | 2.68 [1.42-5.04] | 0.002 |
| NRT bought over the counter | | 24.4 (41/168) | 0.57 [0.39-0.84] | 0.004 |  | 0.67 [0.44-1.01] | 0.056 |  | 0.71 [0.46-1.08] | 0.107 |
| E-cigarettes | | 32.0 (110/344) | 0.86 [0.65-1.14] | 0.286 |  | 1.07 [0.79-1.45] | 0.677 |  | 1.09 [0.80-1.50] | 0.576 |
| Traditional behavioural support | | 36.0 (31/86) | 1.28 [0.77-2.14] | 0.337 |  | 1.28 [0.77-2.11] | 0.338 |  | 1.20 [0.69-2.06] | 0.522 |
| CI, confidence interval. NRT, nicotine replacement therapy. OR, odds ratio.  ^1^Model 1 = multivariable model including all smoking cessation aid variables, but no potential confounders.  ^2^Model 2 = multivariable model including all potential confounders (age, sex, social grade, alcohol consumption, strength of urges to smoke, time since the quit attempt started, number of prior quit attempts in the past year, whether the quit attempt was planned, whether the participant quit abruptly versus gradually, year of the survey, and timing of the Covid-19 pandemic), but no other smoking cessation aid variables.  ^3^Model 3 = fully adjusted multivariable model including all potential confounders and all smoking cessation aid variables.  Each OR and 95% CI is for using the smoking cessation aid in question relative to not using that smoking cessation aid.  ^4^Prescription NRT, varenicline, and bupropion combined. Data are not presented separately for bupropion due to small sample size (n=4). | | | | | | | | | | |
